# Activated p53 with Histone Deacetylase Inhibitor Enhances L-Fucose-Mediated Drug Delivery through Induction of Fucosyltransferase 8 Expression in Hepatocellular Carcinoma Cells

**DOI:** 10.1371/journal.pone.0168355

**Published:** 2016-12-15

**Authors:** Yutaka Okagawa, Kohichi Takada, Yohei Arihara, Shohei Kikuchi, Takahiro Osuga, Hajime Nakamura, Yusuke Kamihara, Naotaka Hayasaka, Makoto Usami, Kazuyuki Murase, Koji Miyanishi, Masayoshi Kobune, Junji Kato

**Affiliations:** Department of Medical Oncology and Hematology, Sapporo Medical University School of Medicine, Sapporo, Japan; University of Hong Kong, HONG KONG

## Abstract

**Background:**

The prognosis of advanced hepatocellular carcinoma (HCC) is dismal, underscoring the need for novel effective treatments. The α1,6-fucosyltransferase (fucosyltransferase 8, FUT8) has been reported to accelerate malignant potential in HCC. Our study aimed to investigate the regulation of FUT8 expression by p53 and develop a novel therapeutic strategy for targeting HCC cells using L-fucose-mediated drug delivery.

**Methods:**

Binding sites for p53 were searched for within the *FUT8* promoter region. FUT8 expression was assessed by immunoblotting. Chromatin immunoprecipitation (ChIP) assays were performed to analyze p53 binding to the *FUT8* promoter. The delivery of Cy5.5-encapsulated L-fucose-liposomes (Fuc-Lip-Cy5.5) to a *Lens Culinaris* agglutinin-reactive fraction of α-fetoprotein (AFP-L3)-expressing HCC cells was analyzed by flow cytometry. The induction of FUT8 by histone deacetylase inhibitor (HDACi) -inducing acetylated -p53 was evaluated by immunoblotting. Flow cytometric analysis was performed to assess whether the activation of p53 by HDACi affected the uptake of Fuc-Lip-Cy5.5 by HCC cells. The cytotoxicity of an L-fucose-bound liposome carrying sorafenib (Fuc-Lip-sorafenib) with HDACi was assessed *in vivo* and *in vitro*.

**Results:**

The knock down of p53 with siRNA led to decreased FUT8 expression. ChIP assays revealed p53 binds to the *FUT8* promoter region. Flow cytometric analyses demonstrated the specific uptake of Fuc-Lip-Cy5.5 into AFP-L3-expressing HCC cells in a p53- and FUT8-dependent manner. HDACi upregulated the uptake of Fuc-Lip-Cy5.5 by HCC cells by increasing FUT8 via acetylated -p53. The addition of a HDACi increased apoptosis induced by Fuc-Lip-sorafenib in HCC cells.

**Conclusions:**

Our findings reveal that *FUT8* is a p53 target gene and suggest that p53 activated by HDACi induces Fuc-Lip-sorafenib uptake by HCC cells, highlighting this pathway as a promising therapeutic intervention for HCC.

## Introduction

Hepatocellular carcinoma (HCC) is the sixth most frequently diagnosed malignancy and the second leading cause of all cancer mortalities worldwide.[1] Most cases of HCC develop from viral infections such as hepatitis B, hepatitis C and non-alcoholic steatohepatitis as a result of chronic liver damage, inflammation, and regeneration.[2,3] Accumulating evidence suggests that malignant transformation of infected hepatocytes could be driven by genetic and epigenetic changes caused by chronic inflammation and DNA damage. Furthermore, proteins derived from hepatitis B and hepatitis C virus-encoded factors directly interact with signaling molecules and accelerate malignant transformation in liver cells.[4]

For patients with HCC, serum α-fetoprotein (AFP) is generally used as a serologic marker. However, AFP has limited sensitivity and specificity for detecting HCC.[5] Recently, the *Lens Culinaris* agglutinin-reactive fraction of α-fetoprotein (AFP-L3) has been shown to be a useful and specific marker for diagnosing HCC.[6] Expression of AFP-L3 has also been demonstrated to correlate with the prognosis of HCC patients.[7]

AFP-L3 is synthesized by α1,6-fucosyltransferase (fucosyltransferase 8, FUT8), the only enzyme responsible for α1,6-linked fucosylation involving the addition of fucose to the innermost GlcNAc residue of an N-linked glycan.[8] FUT8 is overexpressed in several malignancies including lung,[9] colorectal cancers,[10] and HCC.[11,12] Notably, FUT8 levels increase in plasma and liver tissues with progression of hepatocarcinogenesis. Using a FUT8 knockout mouse system, it was shown that the loss of FUT8 inhibits chemical-induced HCC.[13] However, the regulation and function of FUT8 in HCC cells has not been fully elucidated.

The tumor suppressor, p53, is the most commonly mutated gene in human tumors; mutated -p53 facilitates increased proliferation, survival and metastatic potential.[14] Mutations of p53 have been found in approximately 25% of HCC patients.[15] Growing evidence has implicated the p53 pathway in hepatocarcinogenesis and the progression of HCC. Therefore, p53 is an attractive target for HCC therapy. One strategy for targeted cancer therapy is to introduce molecules that activate p53. We have reported that histone deacetylase inhibitor (HDACi) increases p53 transcriptional activities through p53 acetylation.[16,17] Beside its tumor suppressor function, p53 acts as a transcription factor to regulate a number of signaling pathways.[18] However, to date, the relationship between FUT8 and p53 has not been investigated.

In this study, we identified p53-responsive elements within the *FUT8* genomic promoter region and found a new mechanism of FUT8-mediated enhancement of the cellular incorporation of L-fucose-bound liposomes (Fuc-Lip). We demonstrate a new strategy for HCC treatment combining a drug delivery system with increased L-fucose uptake by HDACi induced p53 upregulation.

## Materials and Methods

### Cell culturing

HepG2 cells were obtained from RIKEN BioResource Center (Tsukuba, Japan) and cultured in DMEM (Life Technologies, Carlsbad, CA, USA) supplemented with 10% fetal bovine serum (FBS), L-glutamine, and 1% penicillin–streptomycin. JHH7 and JHH6 cells were purchased from the Japanese Cancer Research Resource Bank (Osaka, Japan) and cultured in Williams E medium (Life Technologies) supplemented with 10% FBS, L-glutamine, and 1% penicillin–streptomycin. These cell lines were maintained in a humidified atmosphere at 37°C and 5% CO_2_. Cell lines were authenticated in 2012 by cytogenesis.

### Reagents

Suberoly anilide hydroxamic acid (SAHA) was purchased from Sigma (St Louis, MO, USA).

### Recombinant adenoviruses and infection

Adenovirus expressing p53 (Ad-p53) and adenovirus-nLacZ (Ad-LacZ) were generated as reported previously.[16] In brief, an E1-deleted, replication defective, recombinant adenovirus was constructed with the use of a modified type 5 adenovirus genome. The cytomegalovirus promoter was used to drive the transcription of human p53 or ß-galactosidase cDNA. For adenovirus gene transfer, cells were incubated with 0–100 multiplicity of infection of adenovirus constructs at 37°C for 24–48 h.

### Western blot analysis

Equal amounts of protein were run on 4%–20% SDS-polyacrylamide gel electrophoresis (PAGE) under reducing conditions and then transferred to polyvinylidene difluoride (PVDF) membranes (Millipore Corp. Billerica, Massachusetts, USA). The blots were probed with the following primary antibodies: anti-p53 (DO-1; Santa Cruz Biotechnology, Santa Cruz, CA, USA), anti-acetyl-p53 (Lys382; Cell Signaling, Beverly, MA, USA), anti-FUT8 (Novus Biologicals, Littleton, CO, USA), and anti-ß-actin (Santa Cruz Biotechnology). Subsequently, the membranes were incubated with goat anti-mouse- or goat anti-rabbit horseradish peroxidase and visualized by enhanced chemiluminescence (GE Healthcare, Amersham, UK). The blots were quantified using LAS-4000UV mini and Multi Gauge software (Fujifilm, Tokyo, Japan).

### Small interfering RNA

One formulation of small interfering RNA (siRNA) directed against p53 (sc-29435) was purchased from Santa Cruz Biotechnology. Two formulations of siRNA directed against FUT8 were obtained from Invitrogen. We used the primers si-FUT8-1 (5’-AGCCAGUAGACCACAUGAUGGAGUCU-3’) and si-FUT8-2 (5’-CAGCUCCAUCAUGUGGUCUACUGCU-3’).

Cells (5 x 10^5^ / well) were grown in 6-well plates, and transfected with siRNA using Lipofectamine RNAiMAX (Invitrogen) according to the manufacturer’s protocol.

### Chromatin immunoprecipitation assays

HepG2 cells were seeded at 5 x 10^6^ cells in 10 cm^2^ plates and transfected with Ad-p53 or Ad-LacZ. Chromatin immunoprecipitation (ChIP) assays were performed 24 h after transfection using the Thermo Piece Agarose ChIP Kit (Thermo Fisher Scientific, Rockford, IL, USA) according to the manufacturer’s protocol. Resuspended DNA was amplified by polymerase chain reaction (PCR) using specific primers for the human *FUT8* promoter binding site (BS) -1 (forward, 5´-ACCTGGCAAGAGAACGACTG-3´; reverse, 5´-TTTAGCTGCCATCCCAAAAC-3´), and BS-2 (forward, 5´-TATCCACCCCACCTCTGACT-3´; reverse, 5´-GCTCAGTTTGGGTGTCATCA -3´).

### Measurement of AFP-L3 values

Total AFP levels of HCC cell lines were measured by enzyme-linked immunosorbent assay (ELISA) at Sapporo Clinical Laboratory Inc. (Sapporo, Hokkaido, Japan). To determine AFP-L3 levels, supernatants of HCC cell lines were immunoprecipitated by lectin (J-Oil Mills, Yokohama, Japan). Lectin-affinity protein was immunoblotted with AFP antibody (sc-8399; Santa Cruz). AFP-L3 levels were quantified by multiplying AFP amounts by the percentage density of the AFP-L3 band.[19]

### Preparation of Cy5.5 and sorafenib encapsulated in liposomes

The preparation of Cy5.5- and sorafenib-carrying liposomes has been described in a previous report.[20] Details can be found in the S1 Text section. Briefly, dipalmitoylphosphatidyl choline, cholesterol, ganglioside, diacetylphosphate, and dipalmitoylphosphatidylethanolamine were mixed at different molar ratios, and cholic acid was added to facilitate micelle formation. The mixture was dissolved in methanol/chloroform (1:1, v/v), and the solvent evaporated at 37°C to produce a lipid film, which was dried under vacuum. For the sorafenib preparation, a human serum albumin (HSA) solution (20 mg/mL, 0.2 N NaOH) containing sorafenib (20 mg/mL) was added to the lipid film and sonicated to obtain uniform micelles, which were then ultrafiltered (10 kD molecular weight cutoff; Amicon PM10, Millipore, Billerica, MA, USA). Hydrophilization treatment and L-fucose conjugation on the surface of liposomes were carried out by methods modified from Yamazaki et al.[21] Aminated L-fucose was conjugated to liposome surfaces through a 3,3’-dithiobis(sulfosuccinimidyl propionate) crosslinker. The average particle size and zeta potential of liposome that were prepared in water were determined by dynamic light scattering spectrophotometry. The lipid concentrations of Fuc-Lip were measured using a Cholesterol E-test Wako Kit (Wako, Tokyo, Japan). Cy5.5-encapsulated Fuc-Lip (Fuc-Lip-Cy5.5) and Fuc-Lip carrying sorafenib (Fuc-Lip-sorafenib), had L-fucose, concentrations of 0 μg/mL (F0-Lip-Cy5.5, F0-Lip-sorafenib), 25 μg/mL (F25-Lip-Cy5.5) and 50 μg/mL (F50-Lip-Cy5.5, F50-Lip-sorafenib), respectively.

### Flow Cytometric analysis of Cy5.5

Cells (1 x 10^5^) were incubated with complete medium for 24 h, after which they were treated with or without SAHA for 6 h, and then incubated with Fuc-Lip-Cy5.5 (lipid concentration: 4 mg/mL) for 30 min. At 30 min post-treatment, the cells were washed twice with PBS. The mean fluorescence intensity was assessed on a FACS calibur with CellQuest software (Becton Dickinson, Franklin Lakes, NJ, USA).

### Growth inhibition assay

HCC cell lines (5 x 10^3^ / well) were seeded onto 96-well plates and, cultured for one day. Cells were incubated with different doses of Fuc-Lip-sorafenib after treatment with or without SAHA for 6 h. After incubation for 2 h, cells were washed twice with PBS, and then resuspended in medium containing serum and antibiotics. After 48 h culture, BrdU cell proliferation assay reagent (Cell Signaling) was added, and the proliferation assay was performed according to the manufacturer’s instructions. Viable cells were expressed as percentage of control.

### HCC xenograft model

Concentrations of Lip-sorafenib or Fuc-Lip-sorafenib via tail vein injection was determined in previous studies.[22–24] For the subcutaneous model, HepG2 cells (2 x 10^6^) were inoculated to create dorsal lesions (mice aged 4 to 6 weeks) that were allowed to grow into tumors of 5 mm in diameter. HCC-bearing mice were treated with F0-Lip-sorafenib (1 mg/kg), or F50-Lip-sorafenib solution (1 mg/kg) via tail vein injection twice per week, with or without SAHA (1 mg/kg) administration (intraperitoneal injection twice per week). At every treatment schedule, tumor volumes were measured according to the formula = length x width^2^/2. For D-mannose pretreatments, in all of the *in vivo* experiments, 5 mg of D-mannose were injected via tail vein 5 min before the administration of agents. These protocols were approved by the Committee on the Ethics of Animal Experiments of Sapporo Medical University School of Medicine. All surgeries were performed under sodium pentobarbital anesthesia, and all efforts were made to minimize suffering.

### Immunohistochemical staining

Tumor tissue samples from HCC patients were immunostained with anti-p53 (Clone DO-7, Dako Japan, Tokyo, Japan) as a primary antibody and visualized using ABC Elite Kits (Linaris, Dossenheim, Germany). Mutated p53 was positive in this assay. We classified the patterns of staining by the method of Gong et al[25] with a minor modification. In brief, scores for the percentage of positive cells were assigned as follows: ≤ 25% of positive cells, 0; 26–50% of positive cells, 1; 51–75% of positive cells, 2, and > 76% of positive cells, 3. Scores for staining intensity were assigned as follows: no staining, 0; light brown, 1; brown, 2; and dark brown, 3. Overall scores ≤ 4 were defined as negative, overall scores >4 but ≤6 were defined as weakly positive, and overall scores > 6 but ≤ 9 were defined as positive. Two independent pathologists examined five random fields (300 mm^2^) of each sample without knowledge of patient outcomes (double blind). An average value of the two scores is presented in the present study.

### Statistics

Results are presented as the mean ± standard deviation for each sample. Differences between pairs of groups were examined by unpaired and paired *t* tests. If two groups could not be considered to be of equal variance, *t* tests with; χ2 and Fisher exact tests were undertaken. For continuous data, the Mann–Whitney *U* test was used. Statistical analyses were two sided and performed using JMP, version 12.0, for Macintosh (SAS, Cary, NC, USA). Results were considered statistically significant if they had a *P*-value < 0.05.

## Results

### p53 regulates FUT8 expression through p53 binding to the *FUT8* promoter region

**Fig 1:**
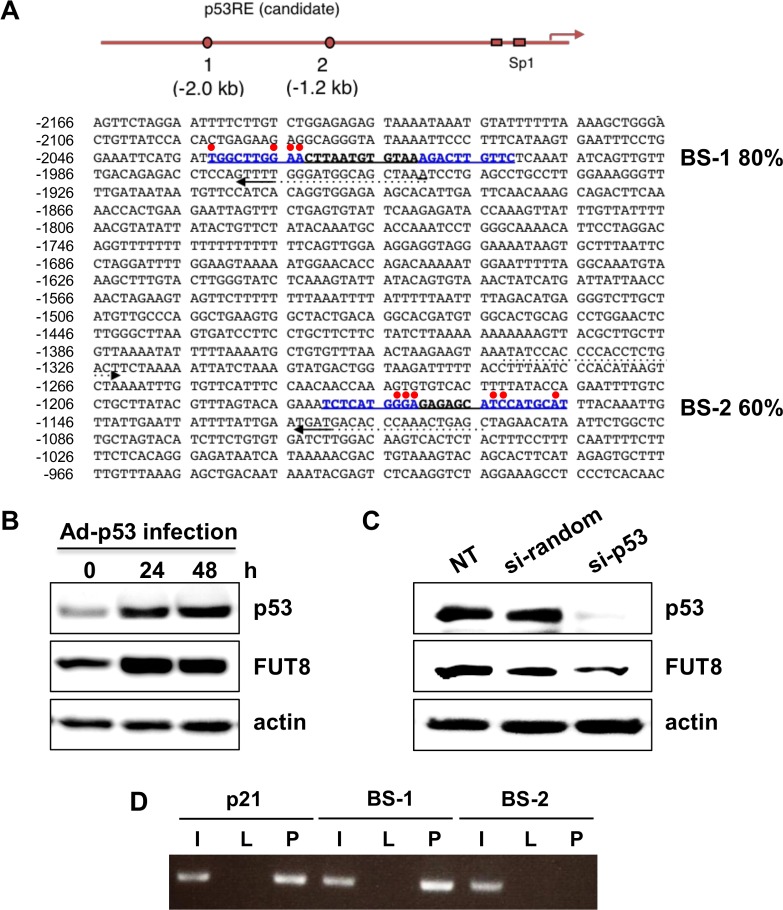
Identification of the p53-DNA binding site within the *FUT8* genomic promoter. (A) Mapping of p53-binding DNA sequences on the *FUT8* genomic locus. Based on DNA sequence results, each candidate shows high homology to the p53-DNA binding consensus sequence (as indicated by%). Red points indicate mismatched residues compared with the consensus sequence, whereas candidate sites are written in blue. (B) Protein expression of p53 and FUT8 in HepG2 cells was analyzed by western blot after 24- and 48-h of infection with Ad-p53. (C) FUT8 protein expression after p53 knock-down in HepG2 cells was determined by western blot. (D) ChIP assay of HepG2 cells infected with Ad-p53 and Ad-LacZ. The PCR product for the p21 binding site of p53 was used as a positive control. I: 10% input, L: Ad-LacZ infection, P: Ad-p53 infection. NT: no treatment. Experiments were carried out in triplicate and repeated at least three times.

We first searched for p53 binding sites within the *FUT8* promoter region using TFBIND software[26] and identified two candidate p53-DNA binding sequences within 2 kb upstream of the human *FUT8* promoter (Fig 1A). These BS were designated BS-1 and BS-2. The BS-1 and BS-2 sequences were 80% and 60% identical to the p53-DNA consensus binding sequence, respectively. To determine if FUT8 induction was p53 dependent, wild type p53 (wt-p53) was transduced into HepG2 cells using an Ad-p53. The expression of p53 was confirmed by immunoblotting. FUT8 protein expression increased after Ad-p53 infection (Fig 1B). Additionally, p53 knocked down with siRNA led to decreased FUT8 protein expression (Fig 1C). A ChIP assay was performed to confirm the direct binding of p53 to these sequences. As shown in Fig 1D, p53 bound to BS-1 but not BS-2. Thus, FUT8 is upregulated by p53 through a conserved p53-DNA binding site in its promoter region, and is transcriptionally activated as one of the p53 target genes.

### Incorporation of L-fucose-bound liposomes into HCC cells is dependent on the p53–FUT8 axis

**Fig 2:**
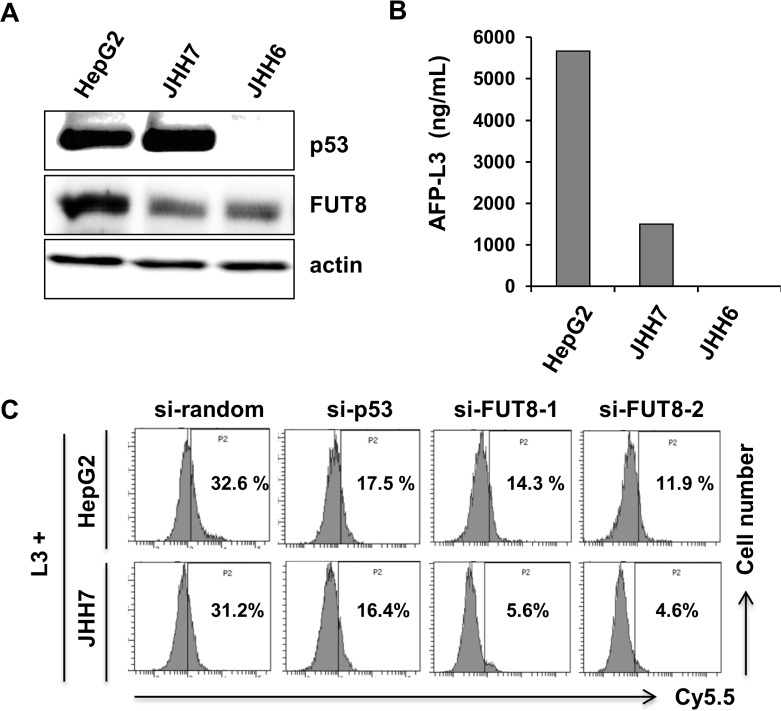
p53 and FUT8 regulate the introduction of Fuc-Lip-Cy5.5 into HCC cells. (A) Expression levels of p53 and FUT8 in HCC cells were examined by western blot. (B) Quantification of AFP-L3 values. (C) Determination of the introduction of Fuc-Lip-Cy5.5 into HCC cells. After siRNA transfection, HepG2 and JHH7 cells were exposed to 1 μM SAHA for 6 h, then treated with F50-Lip-Cy5.5 for 30 min and analyzed by flow cytometry. One siRNA was used for p53 (si-p53) and two were used for FUT8 (si-FUT8-1 and si-FUT8-2). Experiments were carried out in triplicate and repeated at least three times.

The expression levels of p53 and FUT8 in a panel of HCC cell lines were determined by immunoblotting. As shown in Fig 2A, p53 and FUT8 were differentially expressed. HepG2 and JHH7cells harbored wt-p53; JHH6 did not express p53. FUT8 has been reported to catalyze core-fucosylation in AFP-L3 production. AFP-L3 was detected in HepG2 and JHH7 cells (Fig 2B). Regarding JHH6 cells, AFP-L3 could not be measured, despite FUT8 expression, because JHH6 cells do not produce AFP. We have demonstrated that L-fucose is required for CA19-9-producing pancreatic and colorectal cancer cells.[20,27] Accordingly, AFP-L3-expressing HCC cells may take up L-fucose-bound liposomes. We prepared Fuc-Lip-Cy5.5 as reported previously.[20,27] HepG2 and JHH7 cells, which express AFP-L3, took up Cy5.5 in an L-fucose-dependent fashion, but AFP non-producing JHH6 cells did not show any Cy5.5 uptake. (Fig 2C, S1A, S1B and S1C Fig). To verify whether the uptake of Fuc-Lip-Cy5.5 by HCC cells was dependent on p53-FUT8, we knocked down either p53 or FUT8. Knock-down of either p53 or FUT8 by specific siRNAs (S2 Fig), reduced Cy5.5 uptake compared with uptake by random siRNA-transfected cells (Fig 2C). Thus, L-fucose-mediated uptake of Cy5.5 is dependent on p53 and FUT8.

### Induction of FUT8 by p53 activation via HDACi treatment

**Fig 3:**
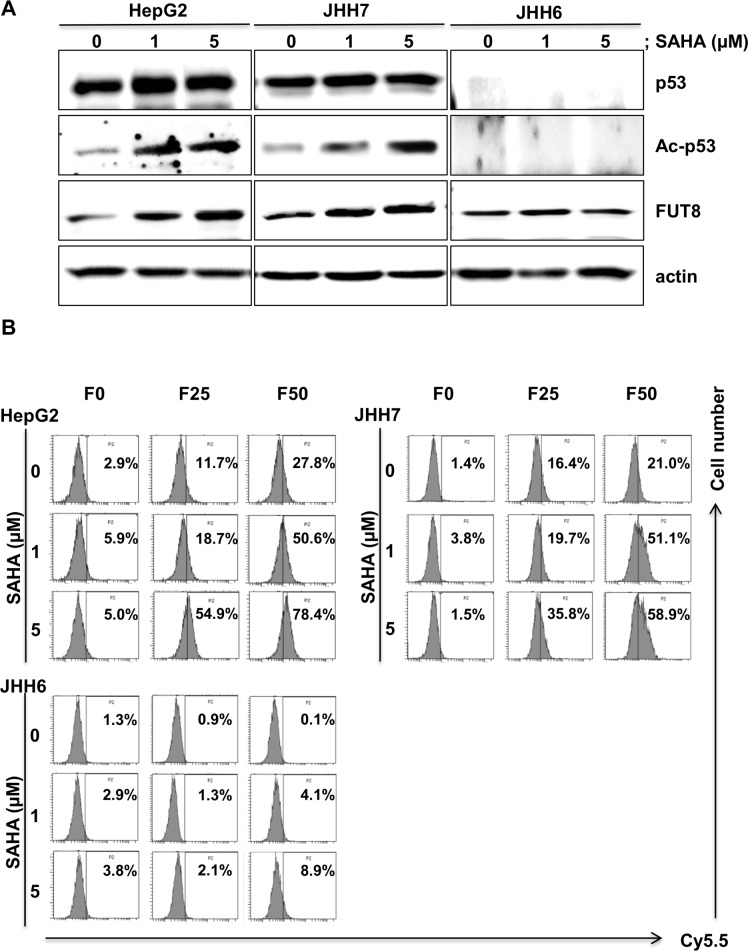
Acetylated-p53 by HDACi up-regulates FUT8 expression and incorporation of Fuc-Lip-Cy5.5 into cells. (A) HepG2,JHH7 and JHH6 cells were exposed to SAHA for 6 h and then the expression levels of p53, Acetylated-p53 (Ac-p53), and FUT8 proteins were analyzed by western blot. (B) Efficacies of incorporation of Fuc-Lip-Cy5.5 into cells. Cells were treated with SAHA (0, 1, 5 μM) for 6 h, then incubated with Fuc-Lip-Cy5.5 for 30 min, and subsequently incorporation of Cy5.5 was analyzed by flow cytometry. The percentages of Cy5.5-positive cells are shown. F0, F0-Lip-Cy5.5: F25, F25-Lip-Cy5.5: F50, F50-Lip-Cy5.5. Experiments were carried out in triplicate and repeated twice.

We previously reported that HDACis, such as sodium butyrate and tricostatin A, enhanced p53 function by lysine acetylation, thereby inducing PIG3 and NOXA, which are pro-apoptotic molecules.[16] Therefore, we used SAHA, a HDACi, to augment FUT8 expression in HCC cell lines. SAHA treatment induced p53 acetylation in a dose-dependent manner (Fig 3A). As expected, expression of FUT8 was increased after a 6-hour treatment of HepG2 cells with SAHA. To determine whether activation of p53 by SAHA affects Fuc-Lip-Cy5.5 incorporation into HCC cells, flow cytometric analysis were conducted. As shown in Fig 3B, the uptake of Cy5.5 was enhanced by SAHA treatment, indicating that SAHA promotes FUT8 expression, leading to L-fucose accumulation.

### Combination treatment of L-fucose liposomes with HDACi induced cytotoxicity more effectively in HCC cells

**Fig 4:**
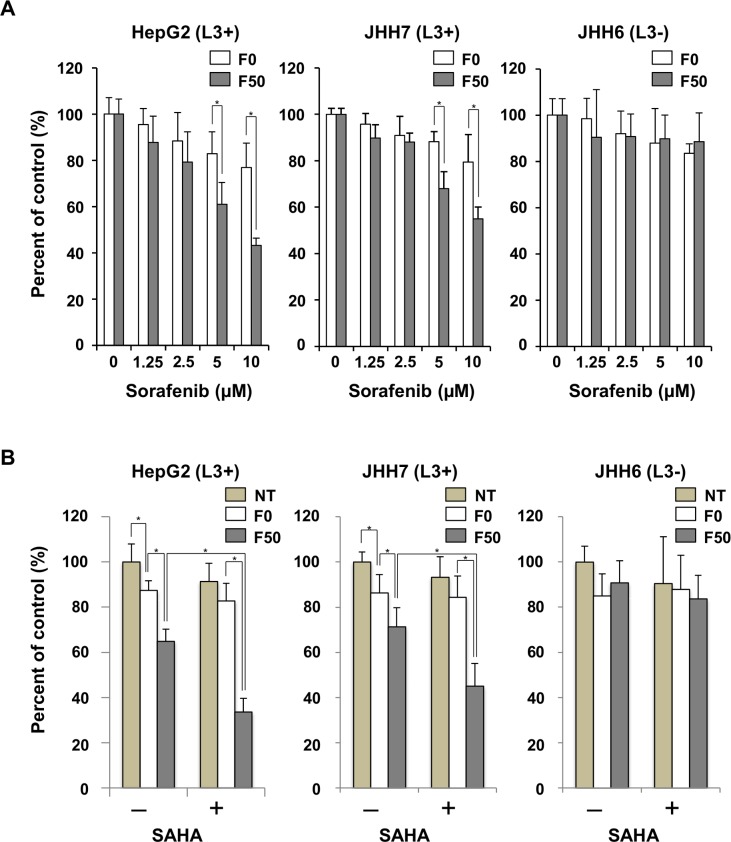
Combination therapy with HDACi enhances the cytotoxicity of Fuc-Lip-sorafenib. (A) AFP-L3 expressing HepG2, and JHH7 cells, and AFP-L3 non-expressing JHH6 cells were treated for 2 h with F0-Lip-sorafenib or F50-Lip-sorafenib. The cells were then, washed, incubated for 48 h, and cell viability was measured by BrdU assay. The percentage of viable cells is shown compared with untreated cells. (B) HCC cells were treated with F0-Lip-sorafenib or F50-Lip-sorafenib (5 μM) for 2 h with or without SAHA (1 μM) and, then washed and incubated for 48 h. Cell viability was measured by BrdU assay. Experiments were carried out in triplicate and repeated three times. NT: no treatment. * *P* < 0.05.

A Fuc-Lip-sorafenib was developed and examined its cytotoxicity and specificity in the presence or absence of SAHA in HCC cells. The concentration of sorafenib was estimated to be 1 mg/mL, and the particle diameters were approximately 120 nm, which is enough to demonstrate enhanced permeability and retention effects (S1 Table). F50-Lip-sorafenib effectively suppressed the growth of AFP-L3 producing HepG2 and JHH-7 cells in a dose-dependent manner compared with F0-Lip-sorafenib (Fig 4A). HCC cell lines were then treated with Fuc-Lip-sorafenib in the presence or absence of SAHA. The sorafenib concentration was chosen to be its IC50. As shown in Fig 4B, even at a low concentration of sorafenib (5 μM), cell proliferation was more effectively inhibited by F50-Lip-sorafenib when combined with SAHA in AFP-L3 producing HepG2 and JHH7 cells. Thus, HDACi increased the cytotoxic effect of Fuc-Lip-sorafenib.

### Combination of HDACi with Fuc-Lip-sorafenib has an additive effect on tumor suppression *in vivo*

**Fig 5:**
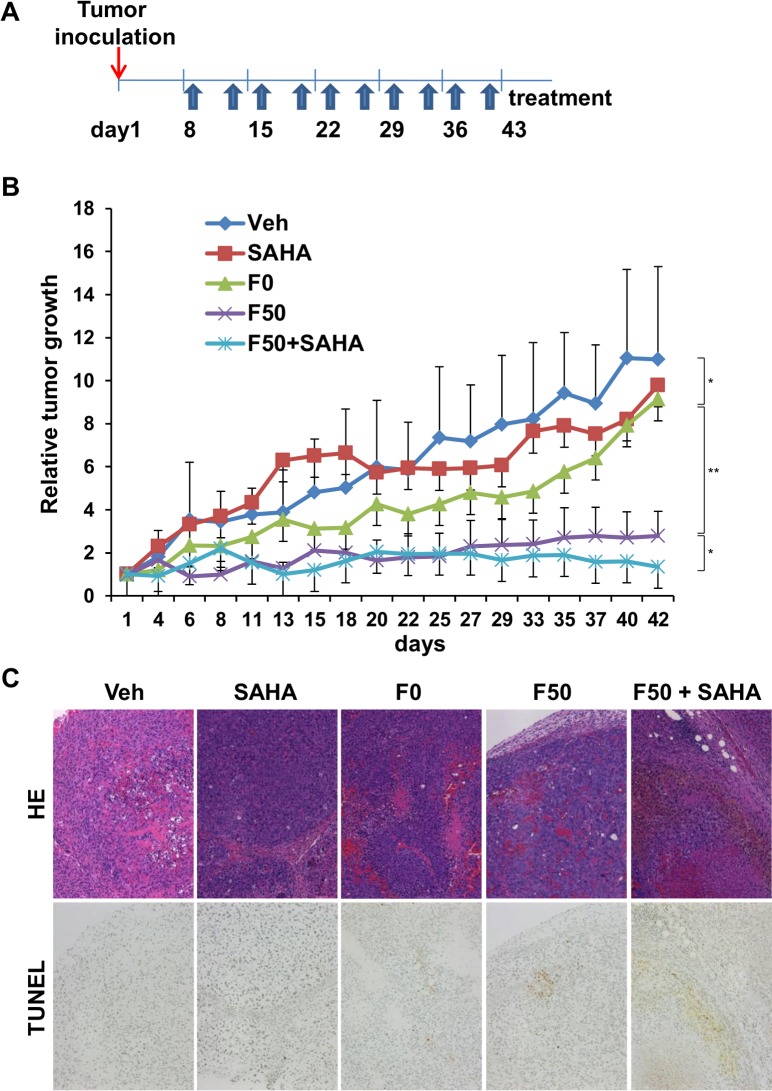
Combination of HDACi and Fuc-Lip-sorafenib augments tumor suppression *in vivo*. (A) Treatment schedule for the HepG2 xenograft mouse model. F0-Lip-sorafenib (1 mg/kg), or F50-Lip-sorafenib solution (1 mg/kg) was administered via tail vein injection twice a week for seven weeks. SAHA (1 mg/kg) was administered daily intraperitoneally. (B) Tumor volumes for each treatment group. At every treatment schedule, tumor volumes were measured according to the formula = length x width^2^ / 2. (C) Tumor tissue was prepared on day 45 after the start of treatment. HE and TUNEL staining in HepG2 tumors are shown. Veh: Vehicle. *P < 0.05, **P < 0.01.

To test the therapeutic potential of Fuc-lip-sorafenib combined with SAHA, we evaluated its ability to inhibit tumor growth *in vivo* using the subcutaneous HepG2 murine xenograft model. Mice were treated with Fuc-Lip-sorafenib twice per week for six weeks in combination with SAHA administration (Fig 5A). As reported previously, D-mannose was administered to enhance specific delivery to tumors.[20] Tumor growth was significantly inhibited by treatment with F50-Lip-sorafenib compared with the vehicle (Fig 5B), F0-Lip-sorafenib, or SAHA groups in HepG2 tumor-bearing mice, suggesting that F50-Lip-sorafenib could specifically and efficiently deliver sorafenib. Furthermore, when SAHA was administered in combination with F50-Lip-sorafenib, the tumor suppressive effected was further enhanced. In hematoxylin-eosin (HE) stains of tumor tissues, many viable cells were observed in the vehicle group. The number of tumor cells decreased in F0-Lip-sorafenib- and F50-Lip-sorafenib–treated mice compared with the vehicle group. However, in mice treated with F50-Lip-sorafenib in combination with SAHA, tumor cells almost completely disappeared. TUNEL staining revealed the greatest number of apoptotic cells occurred in tumors treated with F50-Lip-sorafenib in combination with SAHA, compared to other cohorts (Fig 5C), possibly due to the marked accumulation of sorafenib in tumor tissue. No adverse effects, including body weight changes, were attributable to the administration of either D-mannose or F50-Lip-sorafenib during this study (data not shown).Thus, Fuc-Li-sorafenib combined SAHA had the greatest effect in inhibiting tumor growth in a murine xenograft model of HCC.

### Expression of p53 and AFP-L3 levels in HCC patients

**Fig 6:**
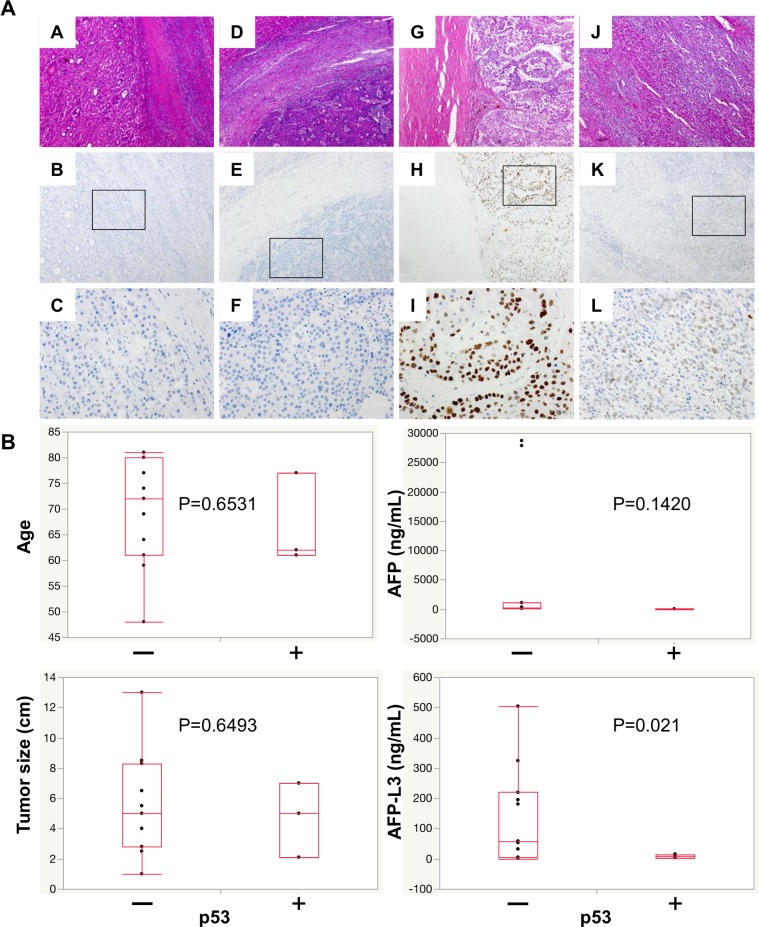
Expression of AFP-L3 correlated with wild-type p53 in HCC patients. (A) HE (A,D,G,J) and IHC (B,C,E,F,H,I,K,L) staining, using anti-p53 antibody, of tumor tissues from HCC patients. The upper and middle row of images were taken at the same magnification (x100), the lower images were taken at higher magnification (x400). (B) Multivariate analysis of p53 expression.

The expression of p53 in tumor tissues from HCC patients was analyzed by immunohistochemistry (IHC) using anti-p53 antibody (Fig 6). Mutant -p53 cells stained positively in this setting. AFP-L3 levels were positively correlated with the expression of mutant p53 in IHC studies (Fig 6, S2 and S3 Tables). These results suggest that wt -p53 expression led to increased AFP-L3 production in HCC patients.

## Discussion

In this study, we identified *FUT8* as a novel target gene for p53 in HCC. FUT8 expression was enhanced by HDACi treatment through the acetylation and activation of p53, thereby enhancing the cytotoxic effect of Fuc-Lip-sorafenib in a murine xenograft model of HCC by increasing its uptake by HCC cells. Thus, a combination strategy using HDACi and Fuc-Lip to encapsulate anticancer drugs could be a promising novel strategy for HCC treatment.

In HCC patients, the positivity of AFP-L3 is around 20–30%; AFP-L3 correlates with poor differentiation, indicating that AFP-L3 may have a prognostic value.[28–30] On the other hand, p53 is mutated in 25% of HCC patients. We demonstrated that AFP-L3 levels were higher in HCC patients with tumors expressing wt -p53 compared to those with tumors showing mutant -p53. Thus, HCC cells produce AFP-L3 through the activation of FUT8 induced by wt-p53. Moreover, overexpression of FUT8 augments the activation of growth factor signaling pathways and thereby promotes cancer cell growth.[13,31]

The expression of p53 was found to occur in poorly differentiated HCC.[32] Such data seem to contradict ours showing that AFP-L3 expression is induced by FUT8, which is activated by wt -p53. However, we believe that FUT8 induction and AFP-L3 production could be stimulated by other signals such as LEF-1/TCF, which has been previously reported to be an inducer of FUT8, in later stages of HCC.[9] In the early stages of HCC, activated p53 may induce FUT8 in response to stress, viral infection, or other cytotoxic events, suggesting that AFP-L3 may be a useful marker for diagnosing early stages of HCC.

In the current study, we found that L-fucose incorporation into HCC cells was mediated by FUT8. We have also recently revealed that pancreatic and colorectal cancer cells directly take up L-fucose and then secrete fucosylated proteins.[20,27] These two previous studies support the present observations. However, it is still uncertain how L-fucose is taken up by HCC cells. The uptake of Fuc-Lip into AFP-L3 expressing HCC cells was inhibited by excess L-fucose completely, but not by other monosaccharides (S1A, S1B and S1D Fig). These results consistently indicated the existence of an L-fucose transporter or receptor rather than a non-specific diffusion system as shown in our previous reports.[20,27] Notably, the knock-down of FUT8 led to reduced Fuc-Lip uptake by AFP-L3 expressing HCC cell lines. This observation supports the association of FUT8 with the uptake of Fuc-Lip. Further investigations to clarify how L-fucose uptake is regulated are warranted.

**Fig 7:**
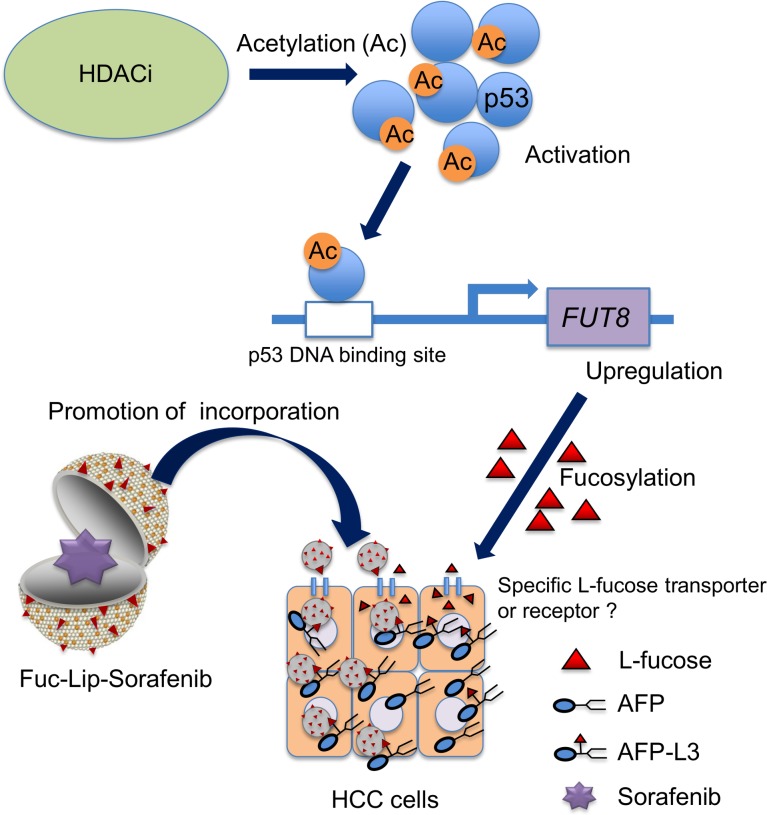
Treatment strategy for AFP-L3 producing HCC using Fuc-Lip-sorafenib. The activation of p53 via its acetylation by HDACi, evokes FUT8 expression. Up-regulated FUT8 induces L-fucose uptake by AFP-L3 producing HCC cells through the L-fucose receptor or transporter. The cytotoxic effect of sorafenib is enhanced by the increased uptake of Fuc-Lip-sorafenib into HCC cells.

HCC has been shown to be resistant to anticancer drugs, although molecular targeting therapies, such as sorafenib, have been recently developed and shown to be effective compared with conventional therapies. One of the reasons for this resistance may be the low expression of p53. So far, only 25% of HCC have been found to express the mutant p53 protein, suggesting that most HCCs may be chemoresistant. Thus, our strategy of targeting HCC cells that produce AFP-L3 looks promising, since it could overcome chemoresistance via an effective delivery system for chemotherapeutic agents and molecular targeting drugs (Fig 7). Furthermore, our strategy may avoid dose-limiting toxicities of sorafenib, such as diarrhea, fatigue and skin toxicity, and improve quality of life in HCC patients.[33]

In conclusion, we have developed a new combination strategy to treat HCC utilizing Fuc-Lip carrying sorafenib, which induces targeted drug accumulation in cancer cells by HDACi.

## Supporting Information

S1 Fig Fucose-mediated introduction of Cy5.5 into HCC cell lines. (PDF)

S2 Fig Suppression of FUT8 by introduction of siRNAs. (PDF)

S1 Table Physicochemical properties of Fuc-liposome sorafenib. (PDF)

S2 Table Patients’ characteristics. (PDF)

S3 Table Univariate and stepwise multivariate analysis of p53 expression. (PDF)

S1 Text This file includes Supporting Materials and Mehods with references. (DOCX)
