# Supplementary figures and images for: Activated p53 with Histone Deacetylase Inhibitor Enhances L-Fucose-Mediated Drug Delivery through Induction of Fucosyltransferase 8 Expression in Hepatocellular Carcinoma Cells
Source: PLoS One. 2016 Dec 15;11(12):e0168355. doi: 10.1371/journal.pone.0168355 (PMC5158067; doi:10.1371/journal.pone.0168355)

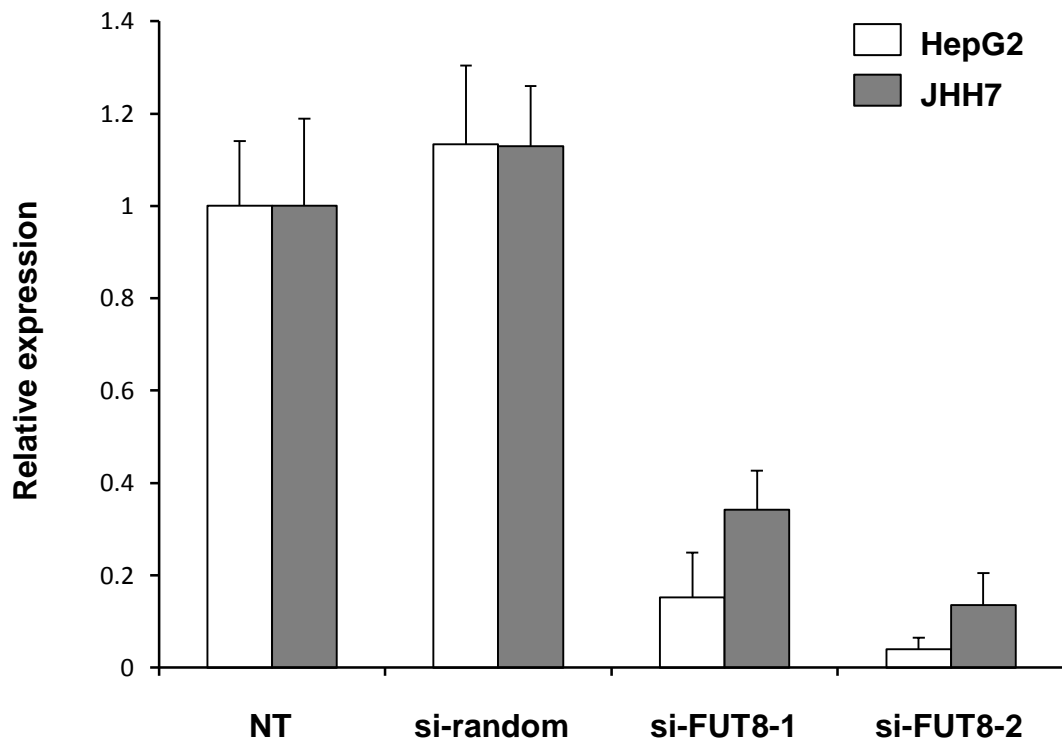

**Supplementary Figure 2. Suppression of *FUT8* by introduction of siRNAs.**

Supplement: S2 Fig — (PDF) [file pone.0168355.s002.pdf]
