# Supplementary material for: Activated p53 with Histone Deacetylase Inhibitor Enhances L-Fucose-Mediated Drug Delivery through Induction of Fucosyltransferase 8 Expression in Hepatocellular Carcinoma Cells
Source: PLoS One. 2016 Dec 15;11(12):e0168355. doi: 10.1371/journal.pone.0168355 (PMC5158067; doi:10.1371/journal.pone.0168355)
Supplement: S1 Table — (PDF) [file pone.0168355.s003.pdf]

Supplementary Table 1. Physicochemical properties of Fuc-liposome sorafenib.

Fucose concentration in conjugating reaction (μg/mL)	0 (F0)	25 (F25)	50 (F50)
Lipid concentration (mg/mL)[a]	37.2	37.7	36.3
Particle size (nm)[b]	121	120	116
z potential (mV)[c]	-79	-82	-84
sorafenib concentration (mM)[d]	1.1	1.1	0.9
sorafenib encapsulation efficiency (%)[e]	5.0	5.0	5.0
sorafenib to lipid weight ratio[f]	0.025	0.025	0.025

[a] Total cholesterol was measured using a Cholesterol E-test Wako Kit.

[b] Encapsulation of Sorafenib into liposomes followed by conversion into Sorafenib 0 in TAPS buffer containing NaCl

[c]Determined with a Malvern Nano-S90.

[d] The amount of Sorafenib was measured by spectrophotometer

[e] Encapsulation efficiency was calculated by the following formula:
Amount of Sorafenib in liposomes/Initial amount of chemicals) × 100

[f] Sorafenib:lipid weight ratio was calculated by following formula:
Sorafenib concentration (mg/mL)/lipid concentration (mg/mL).
