# Supplementary material for: Activated p53 with Histone Deacetylase Inhibitor Enhances L-Fucose-Mediated Drug Delivery through Induction of Fucosyltransferase 8 Expression in Hepatocellular Carcinoma Cells
Source: PLoS One. 2016 Dec 15;11(12):e0168355. doi: 10.1371/journal.pone.0168355 (PMC5158067; doi:10.1371/journal.pone.0168355)
Supplement: S2 Table — (PDF) [file pone.0168355.s004.pdf]

Supplementary Table 2. Patients characteristics

No	Age	sex	Tumor size (cm)	Stage	Pathological findings	Child-Pugh	AFP (ng/mL)	L3 (ng/mL)	Viral infection	P53 staining
1	72	F	1	III	well	5	207.6	1.04	C	-
2	81	F	4	III	mod	5	120.1	58.2	C	-
3	59	M	2.5	II	mod	5	1046	504	C	-
4	74	F	5.5	III	por	5	71.7	32	C	-
5	62	M	5	II	mod	7	25.8	9.29	C	+
6	69	M	6.5	II	well	5	71	4.97	NBNC	-
7	61	F	7	II	mod	5	31.8	15.2	NBNC	+
8	80	M	8.5	II	mod	5	27851	194.9	NBNC	-
9	81	F	8.3	II	mod	5	267.9	180.8	NBNC	-
10	77	M	5	II	por	5	263.6	53.2	NBNC	-
11	48	M	2.8	II	mod	6	1084	324.11	C	-
12	77	M	2.1	II	well	5	38.4	3.22	C	+
13	61	M	4	II	mod	5	357	219.56	C	-
14	64	M	13	III	mod	6	28702	0	C	-
