# Supplementary material for: Activated p53 with Histone Deacetylase Inhibitor Enhances L-Fucose-Mediated Drug Delivery through Induction of Fucosyltransferase 8 Expression in Hepatocellular Carcinoma Cells
Source: PLoS One. 2016 Dec 15;11(12):e0168355. doi: 10.1371/journal.pone.0168355 (PMC5158067; doi:10.1371/journal.pone.0168355)
Supplement: S3 Table — (PDF) [file pone.0168355.s005.pdf]

Supplementary Table 3. Univariate and stepwise multivariate analysis of p53 expression

Characteristics		Number of patients	Univariate analysis			Multivariate analysis
			t	*df*	*p*	
Total		14	0.083	2.894	0.940	
	Male	9				
	Female	5				
Age						
	Median	69	-0.488	3.724	0.653	
	Range	(48 - 81)				
	≤70	7				
	>70	7				
Histology			-0.427	2.562	0.703	
	well	3				
	Moderate	9				
	poor	2				
Tumor size (cm)			-0.488	4.375	0.649	
	≤5	8				
	>5	6				
Tumor stage			-2.390	10.000	0.981	
	0 - II	10				
	III - IV	4				
Viral infection			0.090	12	0.9298	
	C	9				
	NBNC	5				
AFP			-1.594	10.000	0.142	
	Median	235.6				
	Range	25.8 - 28702				
L3-AFP			-2.742	10.100	**0.021**	**0.0064**
	Median	42.6				
	Range	0 - 504				
Child-Pugh			1.243	12	0.237	
	Median	5				
	Range	5 - 7				
