# Supplementary material for: Activated p53 with Histone Deacetylase Inhibitor Enhances L-Fucose-Mediated Drug Delivery through Induction of Fucosyltransferase 8 Expression in Hepatocellular Carcinoma Cells
Source: PLoS One. 2016 Dec 15;11(12):e0168355. doi: 10.1371/journal.pone.0168355 (PMC5158067; doi:10.1371/journal.pone.0168355)
Supplement: S1 Text — (DOCX) [file pone.0168355.s006.docx]

**Supporting Materials and Methods**

**Patients and sample collection**

A total of 14 HCC patients who underwent radical resection for HCC in our hospital were recruited for this study from Jan 1^st^, 2010, to Dec 31^st^, 2014. All patients’ data were obtained from clinical and pathological records, including age, sex, tumor size and depth, lymph node metastasis, and distant metastases. Serum samples were collected from all patients before surgical operation. Serum AFP and AFP-L3 concentrations were determined using a commercially available ELISA kit (Wako, Tokyo, Japan). The postoperative pathological staging of each subject was determined according to the 7^th^ edition of the Union for International Cancer Control tumor-node-metastasis staging system (TNM) for HCC. Tumor tissues were obtained from those patients who gave written informed consent in accordance with the guidelines: (Good Clinical Practice, Ministry of Health and Welfare of Japan). The study was approved by the ethics committees of Sapporo Medical University Hospital, and written informed consent was obtained after detailed explanation in all cases, according to the Declaration of Helsinki.

**Quantitative reverse transcription-PCR**

Total RNA (1 μg) was reverse transcribed (SuperScript VILO cDNA synthesis kit, Life Technologies). Quantitative reverse transcription-PCR (qRT-PCR) was performed using an Applied Biosystems 7300 Real-time PCR system (Applied Biosystems, Foster City, CA, USA). Analysis of target genes was conducted in quadruplicate using the POWER SYBR Green Master Mix (Life Technologies). The primers used for qRT-PCR were as follows: FUT8; forward, 5’-CGTGGA GTCCATCCTGGATATACA-3’, reverse, 5’-CATAAGCAACTCGACAGACCTG-3’, internal standard β-actin: forward, 5′-GGCATCCTCACCCTGAAGTA-3′; reverse, 5′-GAAGGTGTGGTGCCAGATTT-3’.

**Physicochemical characterization of Fuc-Lip-sorafenib**

The average particle sizes and zeta-potentials of liposomes that were prepared in water was determined by dynamic light scattering spectrophotometry (Zetasizer Nano-S90, Malvern, Worcestershire, UK) at 25°C. The instrument was calibrated with standard latex nanoparticles (Malvern, Worcestershire, UK).

**Analysis of lipid concentration**

Lipid concentrations of Lip-sorafenib and Fuc-Lip- sorafenib were measured as total cholesterol in the presence of 0.5% Triton X-100 using a Cholesterol E-test Wako kit. The lipid concentration was calculated from the molar ratio of each lipid (4.5) by the following formula (Eq. (1)):

Lipid concentration (mg/mL) = Cholesterol concentration (mg/mL) x 4.5 (1)
